# Lower CD28+ T cell proportions were associated with CMV-seropositivity in patients with Hashimoto’s thyroiditis

**DOI:** 10.1186/1472-6823-13-34

**Published:** 2013-09-05

**Authors:** Martina Prelog, Jörn Schönlaub, Reinhard Würzner, Christian Koppelstaetter, Giovanni Almanzar, Andrea Brunner, Martin Gasser, Rupert Prommegger, Gabriele Häusler, Klaus Kapelari, Wolfgang Högler

**Affiliations:** 1 Department of Pediatrics, University of Würzburg, Josef-Schneider-Str. 2, Würzburg, Germany; 2 Department of Pediatrics, Medical University Innsbruck, Anichstr. 35, Innsbruck, Austria; 3 Department of Hygiene and Medical Microbiology, Medical University Innsbruck, Fritz-Pregl-Str. 3, Innsbruck, Austria; 4 Department of Internal Medicine, Medical University Innsbruck, Anichstr. 35, Innsbruck, Austria; 5 Department of Pathology, Medical University Innsbruck, Müllerstr. 44, Innsbruck, Austria; 6 Department of Surgery, University of Würzburg, Oberdürrbacherstr. 6, Würzburg, Germany; 7 Department of Surgery, Medical University Innsbruck, Anichstr. 35, Innsbruck, Austria; 8 Department of Pediatrics, Medical University Vienna, Währinger Gürtel 18-20, Vienna, Austria; 9 Department of Endocrinology and Diabetes, Birmingham, Children’s Hospital, Steelhouse Lane, Birmingham, United Kingdom

**Keywords:** Immunosenescence, CD62L, Regulatory T cells, TREC, Telomere

## Abstract

**Background:**

Alterations in the naive T cell subpopulations have been demonstrated in patients with T cell mediated autoimmune disorders, reminiscent of immunological changes found in the elderly during immunosenescence, including the switch from CD45RA + to CD45RO + T cells and decreased thymic function with increased compensatory proliferative mechanisms, partly associated with latent Cytomegalovirus (CMV) infection. The present study was aimed to investigate proportions of lymphocytes, their relation to CMV-seropositivity and the replicative history of CD45RA + expressing T cells in Hashimoto’s thyroiditis (HT, n = 18) and healthy controls (HC, n = 70).

**Methods:**

Proportions of peripheral T cells were investigated by flow cytometry. The replicative history was assessed by T cell receptor excision circles (TRECs) and relative telomere length (RTL). Expression of CD62L was analyzed by immunohistochemistry in thyroid sections. The role of CMV was assessed by serology, ELISPOT assay and in situ hybridization.

**Results:**

Our results demonstrated a significant increase of CD28-negative T cells, associated with CMV-seropositivity in HT patients. HT showed abundant CD45RO + T cells with peripheral loss of CD62L-expressing CD8 + CD45RA + T cells, the latter mainly depending on disease duration. CD62L was expressed in thyroid lymphocyte infiltrations. The diagnosis of HT and within the HT group CMV-seropositivity were the main determinants for the loss of CD28 expression. RTL was not different between HC and HT. HT showed significantly lower TRECs in CD4 + CD45RA + T cells compared to HC.

**Conclusions:**

Patients with HT display a peripheral T cell phenotype reminiscent of findings in elderly persons or other autoimmune disorders. Whether these mechanisms are primary or secondary to the immunological alterations of autoimmune conditions should be investigated in longitudinal studies which may open research on new therapeutic regimes for treatment of HT and associated autoimmune diseases.

## Background

Naive T cell subpopulations are significantly altered in patients at the onset of T cell mediated autoimmune disorders, such as diabetes mellitus type 1 and juvenile idiopathic arthritis (JIA) [1,2]. These alterations are reminiscent of immunological changes found in the elderly during the aging of the immune system (immunosenescence), such as the switch from the CD45RA + to the CD45RO + T cell phenotype and a decrease in thymic function with increased compensatory mechanisms [3,4]. Patients with rheumatoid arthritis and JIA demonstrated increased CD28-negative T cells, an erosion of telomere length as a marker of replicative senescence and a loss of T cell receptor excision circles (TRECs) as a parameter of thymic function and peripheral proliferation of naive T cells [4]. TRECs are stable circular DNA fragments that are created during T cell receptor rearrangement in the thymus [5,6] and have been described to be an indicator of thymic production. TRECs are not replicating during mitosis. Therefore, TREC numbers are as much influenced by peripheral cell turnover as by the influx of newly generated TREC-positive T cells from the thymus.

Latent Cytomegalovirus (CMV) infection was shown to be a driving factor for T cell differentiation [7]. The majority of CMV-specific T cells are included within the CD28-negative T cell subpopulation. CMV-seropositive healthy individuals demonstrated accelerated loss of CD28 expression [8], which was not seen in CMV-seropositive JIA patients [9].

Hashimoto’s thyroiditis (HT) is an organ-specific autoimmune disorder of the thyroid gland associated with diffuse lymphoid infiltration, inflammatory destruction of follicular cells and auto-antibodies against thyroid-specific self-antigens [10-12]. However, the etiopathogenesis of HT is far from being clearly understood [13]. Previous studies have shown significant alterations of the peripheral B and T cell subpopulations in HT [14] with a restricted T-cell-receptor repertoire [15] and a suggested role of CD25+ regulatory T cells in balancing immune tolerance [16-20].

Thus, the present study was aimed to assess whether patients with HT show proportional alterations of the peripheral T cell subpopulations as found in other T cell-mediated autoimmune diseases. In order to investigate the different T cell subpopulations, T cells were characterized by expression of CD3 and either CD4 or CD8, B cells by CD19 and natural killer (NK) cells by CD3-CD16/56+. HLA-DR was used as an activation marker of T cells. CD28 is a co-stimulatory molecule, which is lost through differentiation of T cells. CD45RO mainly defines the memory T cell subset, expression of CD45RA activated naive T cells expressing the CD62L lymphocyte homing factor [21]. High expression of CD25 in combination with CD62L is characteristic for regulatory CD4+ T cells, and in low amounts also for naive T cells.

A further aim was to investigate the replicative history of CD45RA + expressing T cells by assessment of TRECs and relative telomere length (RTL) and the association with CMV-seropositivity. In our study, dilution of TRECs was used to estimate the thymus output and the peripheral proliferative history of the CD45RA-expressing T cell pool, which contains recent thymic emigrants (usually high TREC numbers), pre-existing peripheral, naive T cells (usually low TREC numbers due to peripheral replication and dilution of TRECs) and a small amount of differentiated effector T cells (usually TREC-negative).

## Methods

### Study population

Peripheral blood mononuclear cells (PBMCs) and serological samples were obtained from 18 patients with HT and 70 healthy controls (HC) (Table 1). Diagnosis of HT was based on clinical, serologic and ultrasound findings according to the definitions of the American Thyroid Association. Within the HT group, 6 patients were newly diagnosed (disease duration <6 months). Those otherwise healthy patients presented with either fatigue, hair loss or weight gain and were advised to visit our outpatient Department by their physician. HT patients with other autoimmune diseases (e. g. diabetes type 1), other endocrinological diseases, malignancies, treatments known to influence immunological parameters (e. g. glucocorticoids), immunodeficiency, pregnancy, acute infection in the last two weeks, severe allergies needing local or systemic therapies or administration of vaccines within the last four weeks were not included into the study. Patients were recruited from outpatient clinics at the Departments of Pediatrics, Surgery or Nuclear Medicine, Medical University Innsbruck, Vienna and Wuerzburg in the study period 2011 to 2012. Immunologically healthy HC scheduled for elective surgery (e. g. hernia or plastic surgery) without any therapy, autoimmune disorders, endocrinologic diseases, cancer or other immunological impairments were recruited at the outpatient clinics at the Departments of Pediatrics or Surgery, Medical University Innsbruck.

**Table 1:** Characteristics of the study populations

**Groups (number; male/female)**	**Age (years)**	**Duration of disease (years)**	**TSH (μU/ml)**	**fT3 (pmol/l)**	**fT4 (pmol/l)**	**TPOAb/TRAb/TgAb positive (number)**	**CMV IgG positive/negative (number)**
HC (70; 27/43)	13.7 ± 4.0 (14.6; 5.0 – 28.5)	n. a.	0.9 ± 0.3 (0.8; 0.7 – 1.2)	5.4 ± 1.0 (4.9; 4.7 – 6.6)	14.6 ± 1.9 (15.5; 12.3 – 15.9)	0/0/1	21/49
HT (18; 4/14)	14.1 ± 5.8 (13.6; 4.2 – 34.3)	3.1 ± 2.5 (3.3; 0.3-7.3)	6.9 ± 12.2 (2.1; 0.2 – 50.4) *	5.9 ± 1.2 (5.9; 4.1 – 8.8)	14.34 ± 3.1 (15.0; 8.4 – 19.1)	18/17/18	5/13

Values are given in mean ± standard deviation (median; range).

*Abbreviations*: Healthy controls (HC); Hashimoto’s thyroiditis (HT); thyroid peroxidase antibody (TPOAb); thyrotropin-receptor antibody (TRAb); Tg antibody (TgAb); not applicable (n. a.).

Difference between HT and HC: * p < 0.05.

Serological measurements of thyroid-stimulating hormone (TSH) (normal range: 0.35-3.50 μU/ml), free triiodothyronine (fT3) (3.10-6.50 pmol/l), free thyroxine (fT4) (10.30-21.90 pmol/l), thyroid peroxidase antibody (TPOAb), thyrotropin-receptor antibody (TRAb), and Tg antibodies (TgAb) were performed by ELISA (Roche Diagnostics, Vienna, Austria) according to standard laboratory procedures at the Central Laboratory, University Hospital Innsbruck.

As CMV is well known to drive T cell differentiation and alter proportions of peripheral lymphocyte subpopulations [7-9], HC and HT were separated into CMV IgG seropositive and seronegative subgroups. Anti-CMV IgG and IgM were measured by ELISA (Enzygnost, Dade Behring, Vienna, Austria) according to standard laboratory procedures at the Department of Hygiene and Medical Microbiology, Medical University Innsbruck.

Fourteen HT patients had treatment with levothyroxine (dosage 75–150 μg/day). Thyroidectomy was performed in 7 HT patients due to large nodular goiter in 2 cases and suspect inactive nodules in 5 cases. These 7 patients had been treated with levothyroxine in the last 6 months preceding blood sampling.

All patients or their legal guardians gave their written informed consent to participate in the study. The study was performed according to the Declaration of Helsinki and was approved by the local ethical committee of the Medical University Innsbruck.

### Lymphocyte separation

PBMCs were isolated by using LymphoPrep™ (Axis Shield, Oslo, Norway) according to manufacturer’s instructions as described previously [2]. CD4^+^CD45RA^+^ (naive) T cells were separated by negative selection using a naive CD4^+^ T cell isolation kit (Miltenyi Biotec, Teterow, Germany), magnetic beads and Auto MACS system with sterile columns (Miltenyi Biotec). Purity of separated CD4^+^CD45RA^+^ T cells was checked using 4-colour flow cytometry (FACS-Calibur flow cytometer; Becton Dickinson, Oxford, United Kingdom) and ranged from 97 to 99%.

### Lymphocyte phenotypes

Lymphocytes were characterized by staining with monoclonal mouse antibodies (mAbs) specific for CD4, CD8, CD45RA, CD45RO, CD28, CD25 and CD62L labeled with fluorochromes Fluoresceinisothiocyanat (FITC), Phycoerythrin (PE), Peridinin-Chlorophyll (PerCP) or Allophycocyanin (APC) (all antibodies were purchased from BD Pharmingen, San Jose, California, USA) for 20 min at room temperature in the dark as described previously [2]. Results were expressed as percentage of gated lymphocytes.

### Quantification of TREC numbers

DNA was extracted from separated CD4^+^CD45RA^+^ T cells using QIAamp DNA Mini Kit (Qiagen, Chatsworth, California, USA). In order to remove contaminations, which would interfere with polymerase chain reaction (PCR), DNA was purified by ethanol-precipitation using 0.4M LiCl_2_ and 2.5-fold the volume 100% ethanol at −20°C for 30 min. After centrifugation, the pellet was washed twice with 70% ethanol to remove remaining salts. The pellet was dissolved in nuclease-free water [2].

Signal-joint TREC concentrations were determined by quantitative SYBR-green real-time PCR based on the coding TREC sequence using an iCycler quantitative RT-PCR system (BioRad Laboratories, Hercules, Canada) and log2 dilutions of an internal standard as described previously [2,6]. We designed primers to amplify a DNA fragment 82 bp across the remaining recombination sequence δrec/ψalpha (5′-CAC ATC CCT TTC AAC CAT GCT-3′ and 5′-GCC AGC TGC AGG GTT TAG G-3′) (MWG Biotech AG, Ebersberg, Germany). PCR reaction was run with 0.5 μg DNA, primers (12.5 μM/25 μl) and 12.5 μl SYBR™ Green Supermix (Bio-Rad Laboratories, Hercules, Canada) in a final volume of 25 μl. Each experiment was performed in duplicates. The thermal cycling profile started with 4 minutes at 95°C. Repeating cycles were performed 40 times at 95°C for 30 seconds followed by 62°C for 30 seconds and 72°C for 30 seconds. The process was followed by 1 cycle at 72°C for 30 seconds and finished with 70 cycles at 60°C for 10 seconds. To identify the TREC quantification end products, gelelectrophoresis of amplified TRECs was performed including the plasmid as positive control and a TREC-negative control. To avoid bias by different numbers of naive T cells, TRECs were calculated in relation to CD4 + CD45RA + T cell numbers [22].

### Telomere length analysis

Determination of relative telomere length (RTL) was performed to estimate the individual replicative history of CD45RA-expressing T cells by calculating the ratio of a quantitative PCR reaction product from the same sample using specific primers for telomeres and a single copy gene as described previously [23,24]. Quantitative PCR is the method of choice for determining telomere length in small extractable quantities of DNA, as is the case in our study. Due to limited sample size not all laboratory investigations regarding quantification of lymphocytes, RTL or TRECs could be performed in all patients. In brief, separate PCR experiments were performed for telomere (T) and 36B4 (a single copy gene, S) in 96 well optical reaction plates with 20 μl of each sample. A serially diluted standard was added to each plate. The primer pair sequences for T were: tel1b 5′-CGG TTT GTT TGG GTT TGG GTT TGG GTT TGG GTT TGG GTT-3′ and tel2b 5′-GGC TTG CCT TAC CCT TAC CCT TAC CCT TAC CCT TAC CCT-3′. Sequences for 36B4 were: 36B4 forward 5′-CAG CAA GTG GGA AGG TGT AAT CC-3′ and 36B4 reverse 5′-CCC ATT CTA TCA TCA ACG GGT ACA A-3′. Reagent mixture composition shared by T and S PCR was 15 mM Tris–HCl (pH 8.0), 50 mM KCl, 200 μM dNTP, 1% DMSO, 2.5 mM DTT, SYBR-Green I. Composition specific for the T PCR was 1.5 mM MgCl2, 1.5 Units AmpliTaq Gold DNA polymerase and 450 nM of each telomere specific primer. Composition specific for the S PCR was 3.5 mM MgCl2, 0.75 Units AmpliTaq Gold DNA polymerase, 300 nM 36B4 primer and 500 nM 36B4rev primer. Experiments were carried out on an ABI Prism 7500 Sequence Detector. The thermal cycling profile started with 15 minutes at 93°C to activate polymerase for T PCR and 10 minutes at 95°C to activate polymerase for the S PCR. Repeating cycles were performed 25 (T) and 40 (S) times at 95°C for 15 seconds followed by 56°C for 1 minute [1].

### Immunohistochemistry

CD62L-expressing lymphocytes were identified after fixation of thyroid tissue samples from the 7 thyroidectomized HT patients with 4% neutral-puffered formalin by staining with mouse-anti-human-CD62L antibody (MIB-1 clone; DAKO, Glostrup, Denmark) using an automated immunostainer (Nexes, Ventana Medical Systems, Tucson, AZ, USA) with 3,3-diaminobenzidine tetrahydrochloride (DAB) A chromogen and counterstaining with Hematoxylin and Bluing Reagent. Slides were evaluated semiquantitatively by two independent investigators (MP and AB) counting the number of CD62L + lymphocytes/100 lymphocytes/high power field (HPF) in at least 5 HPFs of inflammatory hot spots. Healthy thyroid tissue sections of 5 donors without HT who were thyroidectomized due to suspect inactive nodules were used as negative controls.

### CMV in situ hybridization

Of the 7 thyroidectomized HT patients, 3 had a positive serology for CMV. Paraffin-embedded samples from HT patients were cut into 3 μm thick sections. Viral DNA was detected using a Fluorescein-conjugated CMV probe (34816 CMV probe) and Novocastra in situ hybridization ISH detection kit (both from Leica Microsystems, London, UK). Hybridization and detection of CMV was performed according to the manufacturer’s recommendations. Slides were counterstained with Mayers Hemalaun. A CMV-infected colon tissue served as a positive control. The 4 CMV-seronegative HT patients served as negative controls.

### CMV ELISPOT

CMV-specific reactivity of lymphocytes was determined in three of the recently diagnosed and two other CMV-seropositive HT patients and five CMV-seropositive HC by using an Interferon-gamma (IFN-γ) ELISPOT analysis, as described previously [25]. In brief, PBMCs were thawed at 5 × 10^5^ cells/well and stimulated with 0.5 μg/ml peptivator CMV pp65 antigen (Miltenyi Biotec, Bergisch-Gladbach, Germany), medium or phytohemagglutinin (PHA) (3 μg/mL; Sigma, Taufkirchen, Germany) in anti-human IFN-γ-precoated silent-screen 96-wells plates (Mabtech) for 18 hours, developed and quantified using C.T.L. ELISPOT reader software (Bonn, Germany). CMV-seronegative donors were used as negative controls. Results of CMV-specific IFN-γ-producing lymphocytes were expressed in spot forming units/10^6^ cells.

### Statistics

The Kolmogorov-Smirnov test was applied to evaluate the normality of distribution of different parameters. Non-parametric Mann–Whitney U test was used to compare HT and HC (SPSS, Version 19.0, Chicago, IL). For multiple comparisons, Bonferroni’s correction was applied. X^2^ test was used to compare dichotome variables between HT and HC, such as sex (male or female) or CMV (IgG seropositive or seronegative). Spearman Rank’s correlation coefficient was used to analyze correlations of T cell subpopulations and age of subjects. To identify independent factors for alterations in T cell proportions, age, disease duration, CMV-seropositivity, sex and HT disease were entered into a multivariate linear regression model. A p < 0.05 was defined statistically significant.

## Results

### Patients

No significant differences were present between the groups regarding age (Table 1). There was no significant age difference between 6 newly diagnosed HT patients (mean 12.3 ± 1.9 years) and the other HT patients (mean age 14.9 ± 7.2 years). In HT, 77.8% were female compared to 61.4% in HC.

There was no significant difference between HT (27.8%) and HC (30.0%) regarding frequency of individuals positive for CMV-specific IgG (Table 1). One HT patient and three HC were concomitantly positive for CMV-specific IgM. Three of 6 newly diagnosed HT patients were CMV IgG positive (IgM negative).

Significantly higher TSH concentrations were found in patients with HT compared to HC. Six HT patients had TSH concentrations (5.4 to 50.4 μU/ml) above the normal range for age three of them belonged to the newly diagnosed HT group. No differences were seen in free serum thyroid hormones (Table 1). Five of 6 hypothyroid HT patients were CMV-seronegative.

### Lymphocyte subpopulations

Absolute lymphocyte counts were significantly lower in HT patients compared to HC (Table 2). In HC, absolute counts of lymphocytes (R = −0.679; p < 0.0001) and proportions (R = −0.556; p < 0.001) negatively correlated with age. These correlations were not seen in HT patients.

**Table 2:** Comparison of lymphocyte subpopulations in HT and HC

**Lymphocyte subpopulation**	**Healthy control (HC)**		**Hashimoto’s thyroiditis (HT)**	
	**Number**	**Mean ± standard deviation (median; range)**	**Number**	**Mean ± standard deviation (median; range)**
**Lymphocytes absolute per μl**	70	2508 ± 763 (2550; 960 – 4200)	18	1917 ± 659 (1794; 665 – 3264)***
**Lymphocytes (% of white blood cells)**	70	33.6 ± 10.9 (33.3; 15.0 – 62.0)	18	33.1 ± 12.5 (34.5; 15.0 – 54.0)
**CD3+ (% of total lymphocytes)**	66	68.3 ± 6.9 (68.0; 47.8 – 82.5)	18	68.8 ± 8.5 (69.2; 43.8 – 80.8)
**CD4+ (% of total lymphocytes)**	66	39.1 ± 6.9 (39.6; 25.8 – 51.1)	18	38.9 ± 7.1 (40.1; 21.5 – 51.6)
**CD8+ (% of total lymphocytes)**	66	27.8 ± 6.4 (26.5; 13.7 – 44.3)	18	24.1 ± 6.2 (23.3; 14.5 – 38.5)***
**CD19+ (% of total lymphocytes)**	66	16.1 ± 4.3 (15.3; 9.2 – 25.9)	18	18.6 ± 8.2 (16.1; 10.0 – 44.4)
**CD3-CD16/56+ (% of total lymphocytes)**	66	10.9 ± 5.9 (9.7; 3.8 – 33.7)	17	7.3 ± 3.5 (6.4; 3.3 – 15.0)
**HLA-DR + (% of CD3+)**	66	5.9 ± 2.8 (5.2; 1.8 – 12.4)	18	6.1 ± 2.5 (5.4; 2.4 – 11.3)
**CD28- (% of CD4+)**	65	1.5 ± 1.3 (1.3; 0.09 – 8.4)	16	1.1 ± 0.9 (0.9; 0.1 – 2.7)
**CD28- (% of CD8+)**	66	11.1 ± 13.7 (3.9; 0.15 – 61.7)	17	19.8 ± 13.3 (14.5; 4.8 – 52.3)***
**CD45RO + (% of CD4+)**	31	35.9 ± 12.9 (36.4; 1.4 – 59.4)	17	46.2 ± 12.9 (47.3; 23.2 – 74.7)**
**CD45RO + (% of CD8+)**	31	19.7 ± 10.6 (17.8; 0.07 – 48.3)	17	29.1 ± 13.2 (27.7; 11.9 – 57.8)***
**CD45RA + CD62L + (% of CD4+)**	64	61.1 ± 14.4 (60.9; 9.9 – 88.2)	17	54.4 ± 18.5 (53.6; 10.6 – 85.3)
**CD45RA + CD62L + (% of CD8+)**	63	61.6 ± 14.9 (64.2; 16.7 – 93.3)	17	51.9 ± 14.9 (55.3; 26.5 – 72.5)**
**CD25 + CD62L + (% of CD4+)**	64	22.8 ± 11.8 (20.8; 4.5 – 78.1)	17	23.6 ± 7.3 (21.5; 14.2 – 39.4)

Difference between HT and HC: *** p < 0.01, ** p < 0.02. Numbers of investigated samples from HC and HT are given in separate columns. For technical reasons not all parameters were investigated in all HC and HT.

**Figure 1:**
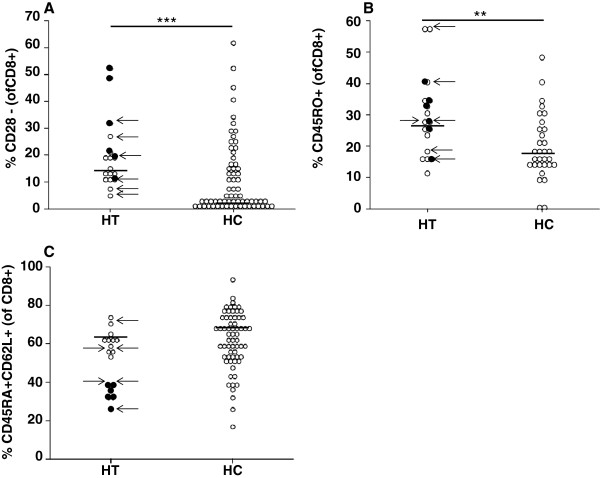
**Percentages of CD28-negative (A), CD45RO + (B) and CD45RA + CD62L + (C) CD8+ T cells patients with Hashimoto’s Thyroiditis (HT) and healthy controls (HC).** Newly diagnosed HT patients are indicated as full circles. Hypothyroid patients are indicated by arrows. Horizontal lines indicate the median. Differences between HT and HC: ** p < 0.02, *** p < 0.01.

HT patients showed significantly lower proportions of CD8+ T cells than HC, higher proportions of CD28-negative CD8+ T cells (Figure 1A) and higher proportions of CD45RO + T cells in both CD4+ and CD8+ T cells (Figure 1B) (Table 2). Newly diagnosed HT patients showed higher proportions of CD8 + CD28- T cells than the other HT patients (p < 0.05) (Figure 1A). Proportions of CD45RA + CD62L + were lower in HT within the CD8+ T cell pool compared to HC (Figure 1C). Newly diagnosed HT patients had significantly lower proportions of CD8 + CD45RA + CD62L + than the other HT patients (p < 0.001) (Figure 1C).

Proportions of CD4 + CD25 + CD62L + T cells correlated with age in HC (R = 0.278; p < 0.05) and HT (R = 0.529; p < 0.05). No correlations between lymphocyte subpopulations and hormone levels or thyroid hormone substitution were seen. We could not find any significant differences between hypo- and euthyroid HT patients (Figure 1A-C).

**Figure 2:**
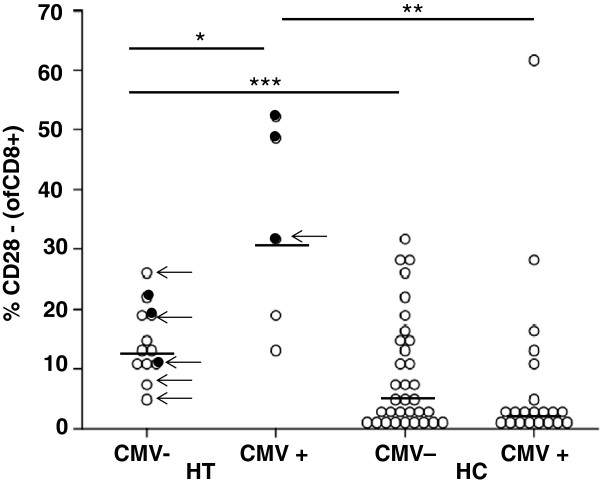
**Percentages of CD28-negative CD8+ T cells in CMV-seropositive (CMV+) or CMV-seronegative (CMV-) patients with Hashimoto’s Thyroiditis (HT) and healthy controls (HC).** Newly diagnosed HT patients are indicated as full circles. Hypothyroid patients are indicated by arrows. Horizontal lines indicate the median. Differences between HT and HC: *** p < 0.05, ** p < 0.02, *** p < 0.01.

CMV-seropositive HC showed higher proportions of CD4 + CD28- T cells compared to CMV-seronegative HC (Table 3). CMV-seropositive HT patients had significantly higher proportions of CD8 + CD28- T cells compared to CMV-negative HT and CMV-seropositive HC (Table 3, Figure 2).

**Table 3:** Comparison of proportions of lymphocyte subpopulations in CMV-seropositive HC and HT

**Lymphocyte subpopulation**	**Healthy controls (HC)**		**Hashimoto’s thyroiditis (HT)**	
	**CMV negative (n = 33)**	**CMV positive (n = 21)**	**CMV negative (n = 12)**	**CMV positive (n = 5)**
**CD28- (% of CD4+)**	1.0 ± 0.8 (0.8; 0.1-3.0)	1.9 ± 1.6 (1.9; 0.2-8.4)##	1.0 ± 0.7 (0.9; 0.3-2.7)	1.4 ± 1.4 (1.4; 0.1-2.6)
**CD28- (% of CD8+)**	9.0 ± 9.4 (5.3; 0.5-32.2)	7.8 ± 14.1 (2.2; 0.2-61.7)	14.5 ± 6.1 (13.3; 4.8-26.4)***	32.6 ± 17.7 (31.4; 12.3-52.3)** #
**CD45RO + (% of CD4+)**	32.5 ± 12.1 (31.5; 4.7-59.4)	30.7 ± 16.9 (38.4; 1.4-41.9)	44.2 ± 14.1 (43.6; 23.2-74.7)***	50.9 ± 9.2 (47.5; 43.4-66.0)**
**CD45RO + (% of CD8+)**	18.7 ± 10.4 (15.7; 0.5-48.3)	14.3 ± 8.6 (15.7; 0.07-21.6)	28.5 ± 15.3 (25.6; 11.9-57.8)***	30.5 ± 6.6 (27.7; 24.8-40.1)*
**CD45RA + CD62L + (% of CD4+)**	61.8 ± 17.6 (65.8; 9.9-88.2)	62.3 ± 12.6 (62.4; 27.5-80.8)	54.8 ± 20.2 (53.3; 10.6-85.3)	53.6 ± 15.5 (60.9; 27.3-66.0)
**CD45RA + CD62L + (% of CD8+)**	64.2 ± 15.0 (68.5; 16.7-83.6)	61.9 ± 15.5 (61.8; 32.2-93.3)	55.1 ± 14.69 (60.1; 26.5-72.5)	43.9 ± 13.9 (39.2; 31.4-61.8)
**CD25 + CD62L + (% of CD4+)**	22.4 ± 11.8 (20.5; 4.5-75.5)	23.7 ± 14.5 (19.7; 10.8-78.1)	25.8 ± 7.5 (24.3; 14.2-39.4)	18.4 ± 2.8 (19.4; 14.7-21.1) #

Values are given in mean ± standard deviation (median; range). Numbers (n) of investigated samples from HC and HT are given in brackets. For technical reasons, lymphocyte populations were not investigated in all HC and HT.

Difference between HT and HC in the CMV-positive or CMV-negative groups: *** p < 0.01, ** p < 0.02, * p < 0.05.

Difference between CMV-positive and CMV-negative individuals in the HC or HT groups: ## p < 0.02, # p < 0.05.

Including HT and HC, age, sex, disease duration and CMV-seropositivity into a linear regression model, the absolute lymphocyte counts (R = 0.493; p < 0.01) and the CD28-negative T cell subpopulations (CD4+: R = 0.504; p < 0.01; CD8+: R = 0.469; p < 0.01) were only influenced by having HT. In the HT group, regression analysis revealed an independent influence of disease duration for lower CD8 + CD45RA + CD62L + T cells (R = 0.809; p < 0.01). CMV-seropositivity was an independent factor for higher proportions of CD28-negative CD8+ T cells (R = 0.744; p < 0.01).

### RTL and TRECs

Relative telomere length was not different between HC and HT (Table 4). Physiological shortening of telomeres with advancing age, as found in HC (R = −0.513; p < 0.0001), was not observed in HT patients. HT showed significantly lower TRECs in CD4 + CD45RA + T cells compared to HC (Table 4).

**Table 4:** Relative telomere length (RTL) and T cell receptor excision circles (TRECs)

**Parameter**	**Healthy control (HC)**		**Hashimoto’s thyroiditis (HT)**	
	**Number**	**Mean ± standard deviation (median; range)**	**Number**	**Mean ± standard deviation (median; range)**
**RTL in CD4 + CD45RA+**	59	1.5 ± 0.9 (1.0; 0.03 – 4.3)	17	1.2 ± 0.4 (1.1; 0.7 – 2.0)
**RTL in CD8 + CD45RA+**	20	1.4 ± 0.6 (1.4; 0.6 – 2.9)	12	1.8 ± 2.9 (1.1; 0.08 – 9.4)
**TRECs/ 1000 CD4 + CD45RA+**	11	2299 ± 205 (2278; 2105 – 2514)	5	554 ± 248 (397; 133 – 730)***
**TRECs/ 1000 CD8 + CD45RA+**	16	1206 ± 1633 (541; 164 – 2615)	4	333 ± 140 (360; 90 – 575)

Numbers of investigated samples from HC and HT are given in separate columns. For technical reasons, lymphocyte populations were not investigated in all HC and HT.

Difference between HT and HC: *** p < 0.01.

### CMV-specific lymphocytes

**Figure 3:**
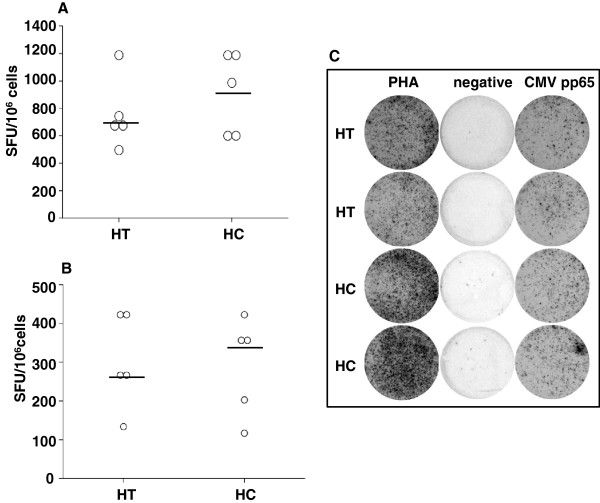
**Interferon-gamma-producing spot-forming-units (SFU) per 10**^**6 **^**cells in patients with Hashimoto’s Thyroiditis (HT) and healthy controls (HC).** Peripheral blood mononuclear cells (PBMCs) were either stimulated with PHA **(A)** or with CMV pp65 antigen **(B)**. A representative example of ELISPOT analysis showed similar results for HT patients and HC **(C)**. Negative controls were unstimulated. Experiments were performed in duplicates.

**Figure 4:**
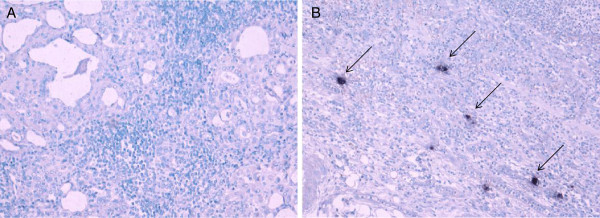
**CMV in situ hybridization in thyroid sections of Hashimoto‘s thyroiditis (HT) patients.** Characteristic infiltrate in a patient with HT with oxyphilic follicular epithelium and surrounding lymphocytes without evidence of CMV DNA **(A)** compared to a positive control of CMV-induced colitis **(B)**. Examples of CMV positive cells are indicated by arrows.

In ELISPOT assay, CMV-seropositive HT patients and HC showed similar numbers of IFN-γ-producing SFU after unspecific stimulation with PHA (Figure 3A,C) or CMV pp65 (Figure 3B,C). CMV in situ hybridization in thyroid tissue of CMV-seropositive HT or HC did not reveal any positive staining for CMV (Figure 4).

### Expression of CD62L in thyroid glands of HT patients

**Figure 5:**
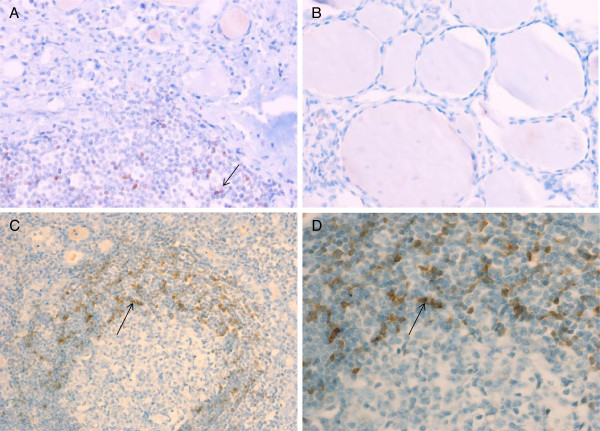
**CD62L-expression in thyroid sections of Hashimoto‘s thyroiditis (HT) patients.** Representative example of CD62L + mononuclear cell infiltrate in the thyroid gland of a patient with HT **(A)** compared to the thyroid gland of a healthy control **(B)** (20× magnification). Representative example of CD62L-expression within a characteristic lymphoid follicle in the thyroid gland of a patient with HT (10× magnification) **(C)**. 40× magnification **(D)**. Arrows indicate examples of CD62L + mononuclear cells.

Staining of CD62L in the thyroid tissue was positive in 5 of 7 HT patients, with a mean of 1.28 cells/100 cells/HPF, 2.56 cells/100 cells/HPF, 0.36 cells/100 cells/HPF, 1.8 cells/100 cells/HPF and 0.08 cells/100 cells/HPF (Figure 5). No difference regarding proportions of peripheral blood T cell subpopulations was found between patients with high or low CD62L + or CD62L-negative staining.

## Discussion

Although it was suggested that HT may mainly result from local autoimmune mechanisms in the thyroid gland [12,13], changes in the peripheral T cell distribution were detected in our study. Our results demonstrated a significant increase of CD28-negative T cells in HT patients, associated with CMV-seropositivity within the CD8+ T cell subpopulation. A role of CD8+ T cells in thyroid inflammation, particularly in HT, was also clearly shown by others [15,26-28]. Our results on CD28 expression differ from a study in 35 children with autoimmune thyroiditis in whom baseline CD28 expression was similar to healthy controls [29]. However, after unspecific stimulation in vitro, CD28 expression decreased in autoimmune thyroiditis patients to a much higher extend as seen in controls in that study [29], which may underline the characteristic down-regulation of CD28 seen in HT patients.

CMV is known to accelerate the increase of CD28-negative T cells [8]. A similar association was also seen in our HT group. These findings support the hypothesis of accelerated differentiation of T cells with latent CMV infections and an association with signs of premature immunosenescence known from patients with autoimmune disorders [4,9,30,31]. Loss of CD28 is also seen as a marker of replicative senescence of the immune system [32]. However, the specificity of CD28-negative CD8+ T cell subpopulations for CMV has to be demonstrated in HT patients, as abundance of CMV-specific CD8+ T cells has been so far shown only for healthy elderly people [33]. In our study, HT patients demonstrated similar CMV-specific lymphocyte reactivity compared to healthy controls. Thus, causality between CMV and loss of CD28-expression cannot be answered by our study, although regression analysis revealed CMV-seropositivity as an independent factor for higher proportions of CD28-negative T cells within the HT group. A primary role of CMV in induction of HT seems unlikely, as a similar prevalence of CMV-specific serum IgG was demonstrated for HT and HC and no expression of CMV-specific DNA was shown in histological sections of CMV-seropositive HT patients. This was in agreement with other studies [34,35] which could not detect DNA of CMV in thyroid specimen of patients with autoimmune thyroid diseases and showed a similar CMV IgG seroprevalance in children with autoimmune thyroid diseases [36]. It seems that CMV is rather involved in a bystander activation of an already altered T cell differentiation which is caused by in part unknown autoimmune mechanisms [37]. Since both HT and CMV are known to be related to immune dysfunction, it is likely that in subjects affected by both conditions the signs of immunological dysregulation are worse than in others.

Regulatory T cells, mostly defined as CD4+ with high expression of CD25, usually increase with age, but obviously have less suppressive function on inflammatory mechanisms in some autoimmune diseases [31]. In our cohort, age was the driving factor for enhancing CD25 + CD62L + T cells. The role of CD25+ T cells in HT has been discussed controversially [38,39]. An increased proportion of CD4 + CD25+ cells was also found in patients with autoimmune thyroiditis irrespective of age [38]. In another study, the proportion of CD4 + CD25+ T cells was not different between newly diagnosed and untreated autoimmune thyroiditis patients compared to HC [39]. In those studies [15,38,39], HT patients appeared to be heterogeneous regarding age, disease activity and thyroid hormone production which makes comparability difficult. In our cohort, the regulatory role of these CD25 + CD62L + T cell fraction is unclear as the use of these markers, CD25 and CD62L, cover both the regulatory and the naive T cell phenotypes.

HT patients demonstrated peripheral loss of CD62L-expressing CD8 + CD45RA + T cells, which was highly significant in newly diagnosed HT patients. In order to further explore the role of CD62L in our HT patients, the expression of CD62L was investigated in histological sections of seven HT patients who underwent surgery. Five HT patients displayed significant CD62L expression in follicular nodules within the afflicted thyroid gland, suggesting a role of CD62L in homing of lymphocytes.

To measure peripheral turn-over of CD45RA-expressing T cells, RTL and TRECs were assessed in some patients in whom DNA of separated CD4 + CD45RA + and CD8 + CD45RA + T cells was available. HT showed lower TREC numbers in CD4 + CD45RA + T cells, with an additional trend towards lower TREC numbers in CD8 + CD45RA + T cells and shorter telomere length in CD8 + CD45RA + T cells. TRECs are influenced by thymic output of recent thymic emigrants and peripheral dilution by proliferation of naive T cells [22]. Since the peripheral CD45RA + T cell pool consists of predominantly naive T cells but also small proportions of differentiated CD28-negative T cells [2], reduction of TRECs may be mostly caused by peripheral replication of CD4 + CD45RA + T cells. However, these findings together with a trend to shorter RTLs and abundance of CD28-negative T cells may support the hypothesis of increased peripheral T cell turn-over and differentiation of peripheral T cells in HT. Whether other mechanisms, such as accelerated switching from CD45RA + to CD45RO+, apoptosis of CD45RA + T cells or distribution of specific T cell subpopulations to secondary lymphatic organs or the thyroid gland (e. g. mediated by homing factors such as CD62L), play a role in diminishing TRECs and in causing accumulation of CD45RO + memory and effector T cells in the periphery remains to be investigated.

## Conclusions

Patients with HT display a peripheral T cell phenotype reminiscent of findings in elderly persons or other autoimmune disorders, such as rheumatoid arthritis [3,40] or JIA [4,31], with increased CD45RO + memory and CD28-negative T cells, increased peripheral replication and altered distribution of T cell proportions, such as CD62L-expressing T cells with accumulation of CD62L + lymphocytes in the thyroid gland. CMV seems to accelerate T cell differentiation by influencing CD28-negative T cell proportions. Whether these mechanisms are primary or secondary to the immunological alterations of autoimmune conditions should be investigated in longitudinal studies which may open research on new therapeutic regimes for treatment of HT and associated autoimmune diseases.

## Competing interests

There are no competing interests.

## Authors’ contributions

MP designed the study and wrote the manuscript. JS recruited the patients, prepared the lymphocytes and performed the flow cytometry analysis. RW performed the CMV ELISAs and interpreted the CMV data. CK performed the telomere length analysis and interpreted the relative telomere length data. GA performed the interpretation of the flow cytometry data and the CMV ELISPOT assays. AB performed the immunhistochemistry, the CMV in situ hybridization and the interpretation of immunhistochemical data. MG interpreted the data and critically discussed the manuscript. RP was involved in patient recruitment and performed the thyreoidectomies. GH performed the recruitment of patients at the University of Vienna and helped in interpretation of clinical data. KK recruited patients and helped in interpretation of clinical data. WH designed the study, interpreted the data and critically discussed the manuscript. All authors read and approved the final manuscript.

## Pre-publication history

The pre-publication history for this paper can be accessed here:

http://www.biomedcentral.com/1472-6823/13/34/prepub
